# Volumetric Single‐Molecule Tracking Inside Subcellular Structures

**DOI:** 10.1002/smll.202509162

**Published:** 2026-01-15

**Authors:** Sam Daly, Joseph E. Chambers, Caroline Jones, Bin Fu, James D. Manton, Joseph S. Beckwith, Stefan J. Marciniak, David C. Gershlick, Steven F. Lee

**Affiliations:** ^1^ Yusuf Hamied Department of Chemistry, Lensfield Road University of Cambridge Cambridge UK; ^2^ Cambridge Institute for Medical Research University of Cambridge Cambridge UK; ^3^ MRC Laboratory of Molecular Biology Francis Crick Avenue Cambridge UK

**Keywords:** diffusion, microscopy, molecular motion, single‐molecule tracking

## Abstract

The non‐covalent interactions that underpin major cellular functions depend on molecular motion within 3D environments. Large depth‐of‐field single‐molecule localization microscopy (3D‐SMLM) methods facilitate these measurements, but their increased optical complexity and bespoke post‐processing pipelines often sacrifice important cellular context. Here, we combine single‐molecule light‐field microscopy (SMLFM) with widefield Fourier light‐field microscopy for correlative volumetric organelle imaging. The instantaneous acquisition of subcellular volumes improves the sensitivity of molecular organization, chemical environment, and diffusion measurements through the use of volumetric sub‐cellular segmentation. We first demonstrate our approach by measuring the molecular organization of a nuclear‐localized HaloTag protein relative to cell nuclei. Next, we characterize the molecular diffusion of the soluble protein, calreticulin, in the context of $\alpha_{1}$‐antitrypsin deficiency, which revealed an increase in heterogeneous motion within endoplasmic reticulum inclusions.

## Graphical abstract

This correlative microscopy approach integrates single‐molecule light‐field microscopy (SMLFM) with widefield Fourier light‐field microscopy (FLFM) to enable 3D single‐molecule tracking within the organelles of living mammalian cells. Instantaneous volumetric segmentation enhances the sensitivity of molecular organization and diffusion measurements, revealing heterogeneous molecular motion within the endoplasmic reticulum of cells expressing disease‐associated $\alpha_{1}$‐antitrypsin polymers.

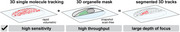

## 1 Introduction

Single‐molecule localization microscopy (SMLM) enables individual biomolecules to be localized with sub‐diffraction resolution [1, 2, 3, 4]. Cells are inherently compartmentalized and a variety of 3D SMLM strategies have emerged to capture molecular organization, biophysical interactions, and diffusion away from the coverslip and within subcellular structures [5, 6, 7, 8].

Most 3D‐SMLM approaches rely on transforming the diffraction‐limited pattern made by a microscope across a defined depth‐of‐field (DoF). This approach is known as point‐spread function (PSF) engineering. Among these methods are astigmatism (≈ 1μm DoF) [9], the double helix PSF (≈ 4μm DoF) [6, 10, 11], and the tetrapod PSF (≈ 8μm DoF) [12, 13]. Alternatively, single‐molecule light‐field microscopy (SMLFM; ≈ 8μm DoF) uses parallax to localize molecules in 3D [14]. SMLFM is based on Fourier light‐field microscopy (FLFM) and enables an order‐of‐magnitude speed improvement at 3D‐SMLM compared to more established methodologies, such as the double helix PSF [15]. Therefore, SMLFM is well‐suited at measuring molecular organization and diffusion in living cellular systems.

Over the years, SMLM has been used extensively to study molecular organization and dynamics close to the coverslip interface, such as the plasma membrane [16, 17], endoplasmic reticulum (ER) [18], and cytoskeleton [19, 20] because their thin and/or flat structures are amenable to the shallow DoF of widefield microscopy. However, measurements of intracellular diffusion *via* single‐molecule tracking (SMT) are limited by axial motion, which causes trajectories to be truncated as molecules exit the focal plane [21]. Large DoF techniques, such SMLFM, enable the quantification of molecular organization and diffusion in complex intracellular architectures, such as the Golgi apparatus. Challenges in 3D‐SMLM include low contrast and the accumulation of background fluorescence from the extended imaging volume [22, 23], both of which can compromise sensitivity. In standard microscopy experiments, subcellular markers in other spectral channels improve sensitivity, but the increased optical complexity and bespoke post‐processing pipelines of 3D‐SMLM mean that vital spatial context is often neglected [24].

**FIGURE 1:**
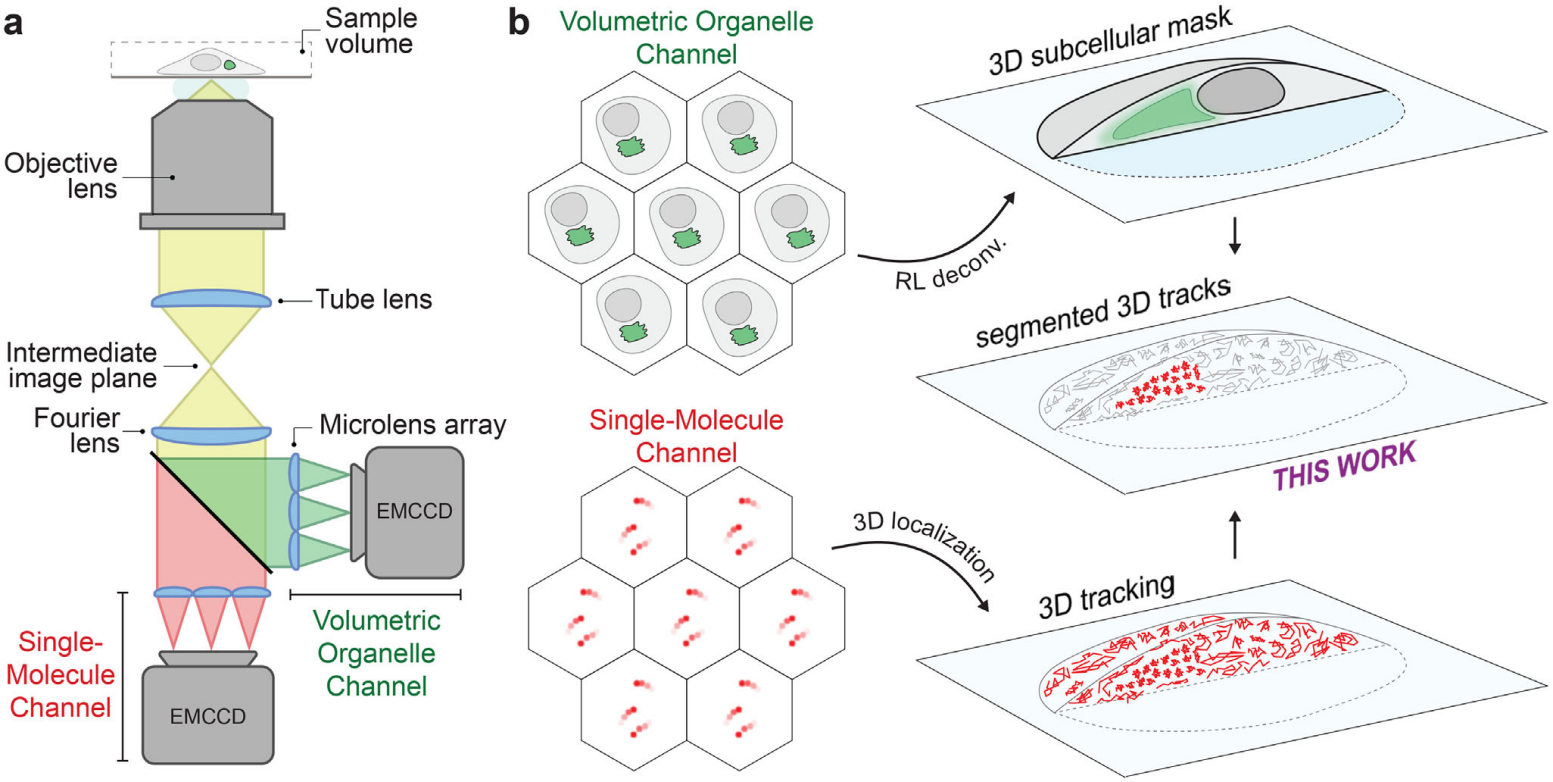
Instantaneous 3D subcellular segmentation of single‐molecule fluorescence using Fourier light‐field microscopy in living cells. (a) Optical schematic of two‐color Fourier light‐field microscopy with key optical elements labeled. Here, one spectral channel is dedicated to widefield instantaneous volumetric imaging (Volumetric Organelle channel; green), while the other is implemented simultaneously for 3D‐SMLM (Single‐Molecule channel; red). (b) Analysis workflow showing 3D reconstruction of the Volumetric Organelle channel with Richardson‐Lucy deconvolution ^[^ ^26^ ^]^ and the Single‐Molecule channel with single‐molecule 3D localization ^[^ ^15^ ^]^. Trajectories are then segmented in 3D according to their localization relative to the subcellular organelle mask for quantitative analysis with improved sensitivity.

The ability to spatially segment molecular localizations in 3D would result in improved sensitivity to molecular organization and diffusion within subcellular compartments. However, optical sectioning requires axial image stacks (i.e., mechanical movement), which is slow compared to the timescales of SMLM experiments, making them prone to photobleaching‐based artifacts [25]. A key functional advantage of FLFM is its instantaneous acquisition of volumetric information without the need for optical sectioning [26, 27, 28]. Conceptually, the challenging question of 3D spatial segmentation is addressed with a FLFM optical platform comprising two imaging channels 1) a widefield channel to instantaneously capture subcellular volumes, and 2) a channel to conduct SMLFM. A simple optical schematic is presented in Figure 1a. Volumetric reconstruction of the widefield imaging channel may be achieved with Richardson–Lucy (RL) deconvolution [26], while SMLFM data can be processed as described previously [14, 15], see Figure 1b. The voxel‐based organelle volume would serve to segment trajectories acquired by SMLFM. In principle, this approach could be applied to track soluble proteins diffusing within a measured volume or across intracellular membranes. Possible research themes that would benefit from increased spatial sensitivity to organelle diffusive sub‐populations include the study of interactions between proteins and their binding partners [29, 30], protein trafficking along the secretory pathway [31], and subcellular membrane organization [32].

Here, we combine SMLFM with widefield FLFM to facilitate the volumetric segmentation of localization data. First, we benchmark the resolution and reconstruction pipeline with realistic simulations and fluorescent microspheres. Next, we confirm its validity in a biological setting by measuring the spatial organization of nuclear‐localized HaloTag proteins in live cell nuclei. Then we characterize the molecular motion of the soluble protein, calreticulin, in the context of $\alpha_{1}$‐antitrypsin deficiency. Using spatial segmentation we revealed an increase in heterogeneous protein motion within endoplasmic reticulum (ER) inclusions, which supports the model of a liquid‐solid phase transition [33, 34].

## 2 Results and Discussion

### 2.1 Concept and Resolution

A Fourier light‐field microscopy platform was integrated into a inverted widefield microscope. In brief, the Fourier lens was positioned in a 4f configuration with the tube lens to produce a collimated beam where a long‐pass dichroic mirror was then positioned. Two identical hexagonal microlens arrays (with seven fully‐illuminated lenses) were placed in the conjugate back focal plane (BFP) of both imaging channels, which focused the imaging volume onto the EMCCDs. In this work, the Volumetric Organelle (VO) channel is represented in green and the Single‐Molecule (SM) channel is represented in red.

**FIGURE 2:**
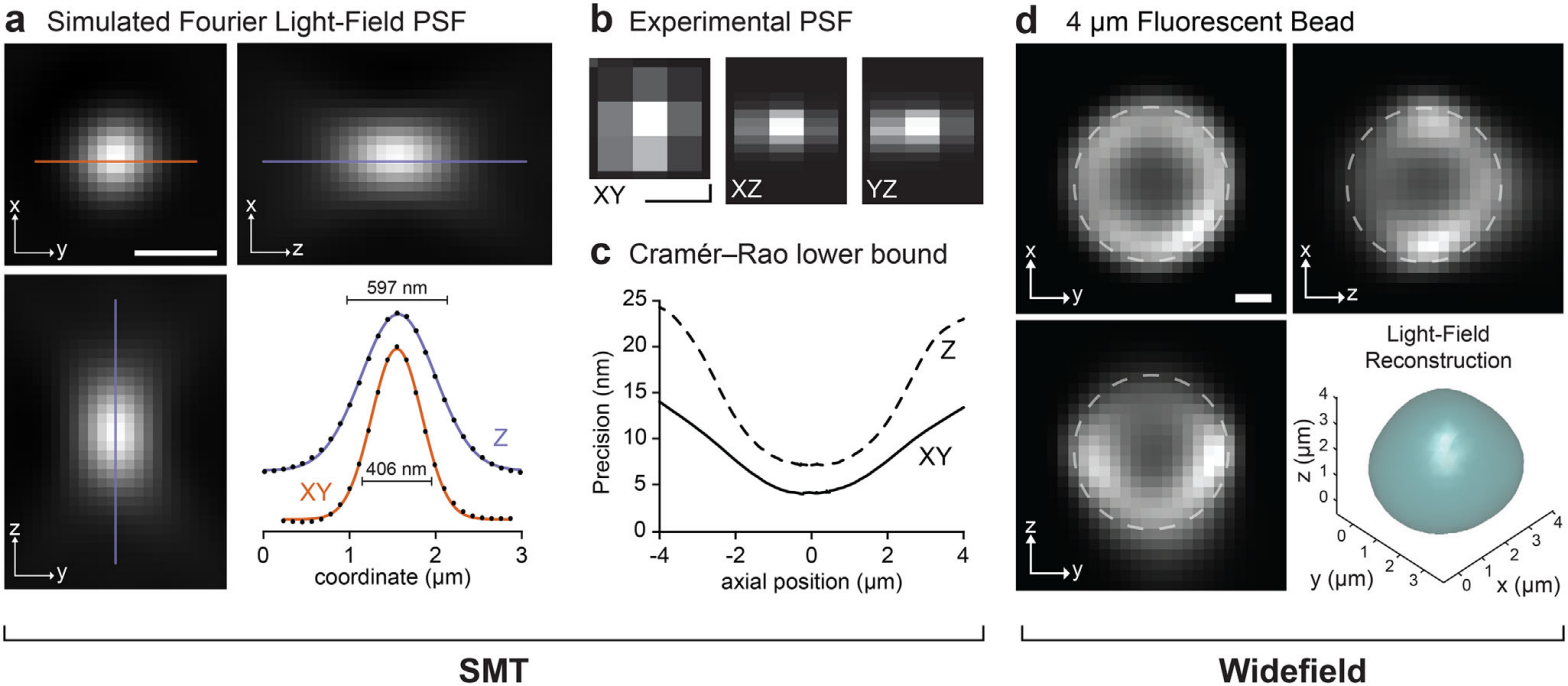
Optical validation. (a) A simulated image of the highly up‐sampled 3D PSF for the optical platform described in this work. Line profiles are presented with FWHM measurements. Scale bar is 1 μm. (b) The experimental 3D PSF measured using a diffraction‐limited fluorescent bead with a pixel size of 266 nm (Nyquist sampling criterion). Scale bar is 0.5 μm. (c) Simulated Cramér‐Rao lower bound describing the precision obtained for a single molecule at 4000 detected photons in 3D. (d) The 3D reconstruction of a ≈4 μm fluorescent bead in glycerol. Superimposed circle has a diameter of 4 μm. Scale bar is 1 μm.

The single‐molecule sensitivity and isotropic nanometer 3D resolution of the SM channel has been previously reported [15]. Specifically, a resolution of ≤ 25nm was demonstrated for fluorescent puncta of 3000 detected photons. This corresponds to the median signal detected from a typical single‐molecule fluorophore, such as AF647, on this platform. To validate the VO channel for high‐magnification volumetric imaging inside living cells we used a combination of realistic simulations and immobilized fluorescent beads. Realistic microscopy datasets were simulated as described in [15] using empirically‐determined optical parameters, but with an artificially high sampling rate and brightness. This enabled the evaluation of the 3D PSF produced by RL deconvolution, see Figure 2a. The full‐width half‐maxima of the lateral (xy) and axial (z) components of the PSF were determined to be 406 and 597 nm, respectively. The equivalent experimental PSF (at a lower sampling rate) is presented in Figure 2b. These values quantify the smallest volumetric spatial scale that can be distinguished with the FLFM configuration. The resolution for SMT was also quantified using the Cramér‐Rao lower bound as <25 nm across the whole 8μm DoF at 4000 detected photons, as shown in Figure 2c. Finally, 4μm fluorescent beads were imaged to confirm the accurate evaluation of particle sizes using the VO channel at the experimental sampling rate (266 nm pixel size), see Figure 2d. The superimposed circle of 4μm diameter and accompanying 3D volume demonstrates the accurate retrieval of particle size and shape for a known geometry.

These validation steps demonstrate that the seven‐lens VO channel can accurately retrieve sub‐micron‐scale spatial structures of a known geometry (fluorescent microspheres) in 3D. This is sufficient for many subcellular organelles, such as the nucleus (≈ 10μm), Golgi apparatus (0.5–2μm) and mitochondrion (1–2μm). Although for smaller cellular structures, such as endoplasmic reticulum tubules (<100 nm), the resolution of this optical platform (≈400–600 nm) would lead to an overestimation of organelle volumes. Nevertheless, the resulting voxel‐based organelle volume would still effectively enrich for diffusive behavior within these subcellular compartments. Conceptually, optical alterations and improvements to fluorescent tags could improve the achievable resolution, but this would only improve the classification of trajectories at the boundaries of the segmented volume.

### 2.2 Segmentation of Nuclear‐Localized Proteins

**FIGURE 3:**
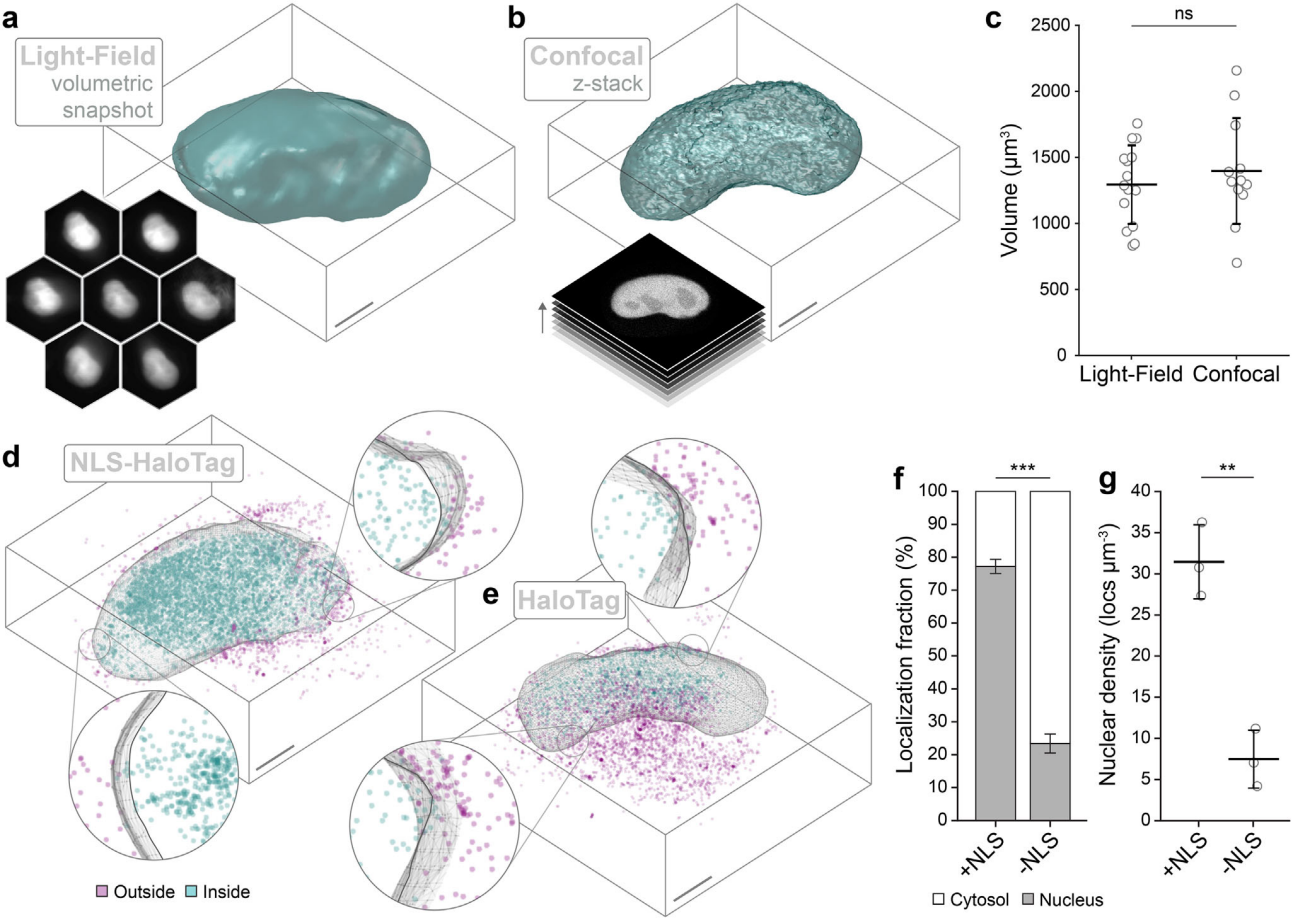
Categorizing 3D localizations according to nuclear location. (a) Representative isosurface of a HeLa cell nucleus by expression of EGFP genetically fused to a nuclear localization signal (NLS‐EGFP). Volume captured instantaneously via Fourier light‐field microscopy (FLFM, see insert, 20 μm field‐of‐view). (b) Representative isosurface of a HeLa cell nucleus under the same conditions via 43 z‐planes using confocal microscopy (insert). Bounding box is ≈10 μm. (c) Nuclear volumes captured by FLFM corroborate those captured by confocal z‐scans. (d) Representative 3D‐SMLM point cloud of NLS‐HaloTag, labeled with ${\text{PA-JF}}_{\text{646}}$. Localisations categorized according to appearance inside (teal) or outside (magenta) of the nuclear volume (NLS‐EGFP). Insert shows cross‐sections of nuclear boundary to demonstrate spatial segmentation. (e) Representative 3D‐SMLM point cloud of cytosolic HaloTag (no NLS), labeled and categorized as in (d). (f) Proportion of NLS‐HaloTag (N = 15 cells, 3 repeats) and HaloTag localizations (N = 13 cells, 3 repeats) appearing inside and outside of the nucleus. (g) Density of NLS‐HaloTag and HaloTag localizations appearing inside the nucleus relative to nuclear volume. Unless stated otherwise, data represents the average value across three biological repeats where error bars represent the standard deviation. For significance t‐tests: *p < 0.05; **p < 0.01; ***p < 0.001. Scale bars are 2 μm.

To validate the VO channel in a biological context it was used to visualize the nuclei of live HeLa cells expressing a nuclear localization signal fused to EGFP (NLS‐EGFP). Nuclear volumes were acquired at an exposure time of 20 ms and averaged over 2000 frames to improve the signal‐to‐noise ratio, see Figure 3a. The 3D volume measurements obtained via RL deconvolution closely corroborated those acquired through 40‐step z‐stacks using confocal microscopy, see Figure 3b,c. This validated the snapshot volumetric imaging pipeline for a biological setting, which is limited by lower SNR and more complex cellular morphologies compared to fluorescent beads.

Next, the VO and SM channels were implemented simultaneously to visualize cells co‐expressing NLS‐HaloTag and NLS‐EGFP proteins, with the HaloTag labeled using ${\text{PA-JF}}_{\text{646}}$ (see Materials and Methods/Note S1). Laser illumination at 640, 488, and 405 nm enabled the acquisition of nuclear volumes in the VO channel and stochastic single‐molecule fluorescence in the SM channel. Post‐processing resulted in a 3D point cloud (NLS‐HaloTag) alongside an isosurface corresponding to the nucleus (NLS‐EGFP), as shown in Figure 3d. The 3D localizations were classified according to their appearance inside (teal) or outside (magenta) of the nucleus, which confirmed nuclear enrichment. The experiment was then repeated using cells co‐expressing a cytosolic HaloTag protein (no NLS) alongside the NLS‐EGFP. A representative 3D point cloud and nuclear isosurface is shown in Figure 3e, which now shows exclusion of localizations from the nuclear volume. The average fraction of molecules that co‐localized with the nuclear volume was quantified at 77% for NLS‐HaloTag, which dropped to 22% for the cytosolic HaloTag, see Figure 3f. Similarly, the density of localizations within the nucleus was fourfold higher for NLS‐HaloTag compared to the cytosolic HaloTag condition, see Figure 3g. These results indicate that the VO channel can precisely capture nuclear volumes in living cells while providing concurrent correlation with 3D‐SMLM measurements.

### 2.3 Measuring Heterogeneous Motion in the ER

Approximately 25% of all proteins transverse the secretory pathway [35]. These secretory proteins fold within the endoplasmic reticulum (ER), a process that relies on the molecular motion of both the nascent polypeptide chains and ER‐resident folding factors. Disruption of this process, leading to protein misfolding, is a hallmark of many human diseases [36]. In $\alpha_{1}$‐antitrypsin deficiency, the pathogenic (Z) variant aberrantly assembles into polymers within the ER of hepatocytes [37]. Cells expressing Z‐$\alpha_{1}$‐antitrypsin display a range of ER morphologies, including the typical reticular network but also fragmented compartments, known as inclusions. It has been hypothesized that a protein phase transition occurs within ER inclusions, which forms a polymer matrix that restricts molecular motion [33, 34]. SMT has been used to observe the solidification of Z‐$\alpha_{1}$‐antitrypsin, but experiments were restricted to the reticular ER network due to a shallow DoF. Here, we perform volumetric SMT of the soluble, ER‐localized chaperone protein, calreticulin, within individual ER inclusions for the first time and reveal heterogeneous molecular motion that is consistent with the formation of a solid protein polymer network.

**FIGURE 4:**
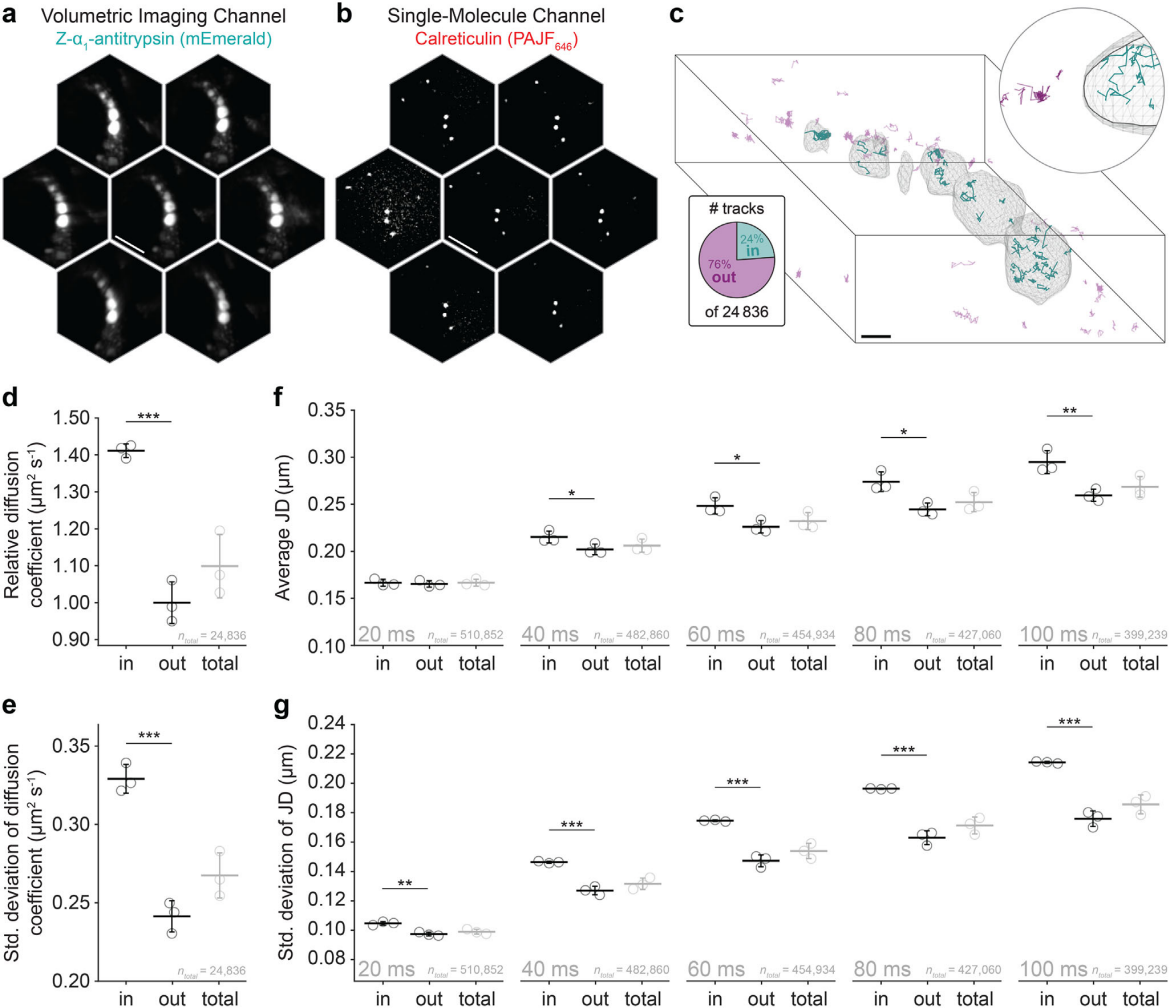
Volumetric SMT with segmentation reveals heterogeneous diffusion within the diseased ER. (a) Representative light‐field image showing several ER inclusions by the expression of Z‐$\alpha_{1}$‐antitrypsin genetically fused to mEmerald. Scale bar represents 10 μm. (b) Representative single‐molecule light‐field image of the soluble ER protein, calreticulin, which was genetically fused to HaloTag protein and labeled with ${\text{PA-JF}}_{\text{646}}$. (c) Single‐molecule trajectories of calreticulin segmented by the 3D reconstruction of Z‐$\alpha_{1}$‐antitrypsin from the mEmerald channel. Teal and magenta tracks correspond to inside and outside tracks, respectively. Expanded view is 5 μm across. Representative of 2000 frames. (d) Average diffusion coefficient (mean square displacement analysis) of calreticulin inside and outside of ER inclusions, and without segmentation (total). (e) Standard deviation of diffusion coefficients among trajectories within each biological repeat (N = 3). (f) Average 3D jump distance inside and outside of ER inclusions, and without segmentation (total), across five lag times (Δ t = 20, 40, 60, 80, and 100 ms). (g) Standard deviation of the 3D jump distances across five lag times (Δ t = 20, 40, 60, 80, and 100 ms) within each biological repeat (N = 3). Unless otherwise stated, error bars indicate the standard deviation across three biological repeats (totalling 19 cells or 24,836 tracks). For significance t‐tests: *p < 0.05; **p < 0.01; ***p < 0.001. Sample size indicated by $n_{\text{tot}}$. Scale bar represents 5 μm.

The VO and SM channels were implemented simultaneously to visualize living CHO‐K1 cells expressing Z‐$\alpha_{1}$‐antitrypsin fused to mEmerald and calreticulin fused to HaloTag. The HaloTag protein was labelled with ${\text{PA-JF}}_{\text{646}}$ to facilitate the visualization of single‐molecule diffusion alongside widefield fluorescence from the ER inclusions, see Figure 4a,b (Movie S1). Prior to image acquisition, the spectral channels were calibrated using multi‐color sub‐diffraction fluorescent beads. During post‐processing, the bead images were super‐localized and a transformation matrix was computed to achieve accurate spectral registration. For volumetric reconstruction, the mEmerald channel first underwent background subtraction and temporal averaging across 2000 frames to minimize reconstruction artifacts that can arise from noise and heterogeneous background fluorescence. RL deconvolution was then implemented using 30 iterations, which was found to best retrieve structural features while minimising noise‐based artifacts. The single‐molecule channel was then super‐localized in 3D and temporally grouped into trajectories, as described previously [15]. The volumetric reconstruction of Z‐$\alpha_{1}$‐antitrypsin was then used to spatially segment the calreticulin trajectories according to appearance inside or outside of ER inclusions, see Figure 4c. A total of 19 cells were imaged across three biological repeats with an exposure time of 20 ms for 20,000 frames (≈7 min) to mitigate phototoxic effects. A total of 24,836 tracks were detected, with an average length of 19.3 points per track. All samples were illuminated using an inclined excitation geometry (HILO) to reduce background fluorescence from the extended volume and improve contrast [15].

Mean square displacement analysis was used to evaluate the diffusion coefficient of calreticulin inside ($D_{\text{in}}$) and outside ($D_{\text{out}}$) of ER inclusions, see Figure 4d. Diffusion within ER inclusions, $D_{\text{in}}$, was observed to be 1.44× faster than outside, $D_{\text{out}}$. We posit that this slower observed relative diffusion outside of ER inclusions is attributed to confinement within the tubular ER network [33, 34]. Without segmentation ($D_{\text{total}}$), this significant difference would have been concealed within a broader distribution. The standard deviation between tracks within each biological repeat was also 1.3× greater for $D_{\text{in}}$ at $0.32\;\mu m^{2}\;s^{-1}$ compared to $D_{\text{out}}$ at $0.24\;\mu m^{2}\;s^{-1}$, as shown in Figure 4e. Jump distance (JD) analysis was then used to examine calreticulin motion on a per‐step basis rather than per track, see Figure 4f and Note S2. Over a time lag of 20 ms (sequential frames) ${\text{JD}}_{\text{in}}=$ 0.167 ± 0.004μm and ${\text{JD}}_{\text{out}}=$ 0.164 ± 0.003μm, which suggests similar modes of molecular motion over short time scales at the resolution of the optical platform. Differences emerge as the time lag was evaluated from 20 to 100 ms (a five frame interval), where now ${\text{JD}}_{\text{in}}=$ 0.30 ± 0.01μm and ${\text{JD}}_{\text{out}}=$ 0.26 ± 0.01μm. This indicates that the average distance moved by calreticulin is greater inside ER inclusions over longer durations. Furthermore, the standard deviation of JDs within each sample in presented in Figure 4g. These data show that for a lag time of 20 ms, the standard deviation in ${\text{JD}}_{\text{in}}$ was 0.105μm, which doubled to 0.214μm for a lag time of 100 ms. Meanwhile, for a lag time of 20 ms, the standard deviation in ${\text{JD}}_{\text{out}}$ was 0.097μm and 0.176μm at 100 ms. Further quantitative details of SMLM datasets can be found in Note S3.

This broader range of diffusive motion aligns with previous reports suggesting the presence of a low‐mobility polymer matrix within ER inclusions, which is thought to arise from a liquid–solid phase transition [33, 34]. Importantly, no correlation was observed when diffusion coefficient was evaluated as a function of inclusion size (see Note S4), which lends support to the interpretation that this diffusive heterogeneity is the result of local confinement within the Z‐$\alpha_{1}$‐antitrypsin polymer matrix, plausibly through both passive trapping by macromolecular crowding and/or dynamic chaperone‐substrate interactions within the low‐mobility polymer matrix. Similarly, the diffusion coefficient of calreticulin within ER inclusions was analysed as a function of inclusion brightness (used as a proxy for polymer density), but no clear trend was observed (see Note S4).

Although volumetric segmentation improved the sensitivity of 3D‐SMT to molecular motion within ER inclusions, key limitations of this imaging approach are as follows: (i) The resolution of the VO channel is approximately an order‐of‐magnitude lower than that achieved in the SM channel and may lead to the accumulation of some false positive trajectories at the segmented boundary; (ii) The distribution of signal across seven perspective views may constrain photon budget and cause difficulties in low‐signal regimes; (iii) Chromatic aberration necessitates additional experimental and computational correction steps to align channels; (iv) The use of an additional spectral channel may limit spectral multiplexing and increase the risk of phototoxic effects from the use of additional lasers; and (v) The precise physical alignment of two detectors is required unless both channels are incident upon a single detector.

Contrast in SMLM is fundamentally constrained by the photophysical properties of fluorophores (i.e., photon budget). This challenge is amplified in SMT experiments, in which emitted photons are distributed across sequential frames. For SMLFM this is coupled with the division of photons across multiple perspective views. In signal‐limited regimes an optical configuration with three illuminated microlenses would boost SNR and spatial precision (higher effective NA) but across a shallower DoF [14]. In this work, a seven‐lens configuration was implemented to capture entire ER inclusions. However, this was at the expense of poorer SNR, which necessitated the use of EMCCDs with a maximum frame rate of 50 fps (20 ms exposure) and read noise of below 1 electron. Furthermore, we implemented SMT and volumetric segmentation using the 488 and 640 nm spectral channels to prevent fluorescence bleed‐through and maximise SNR in the single‐molecule channel. In principle more spectral channels could be accommodated through the optimisation of emission filters and excitation strategies to enable SMT across multiple sub‐cellular compartments. By balancing speed and sensitivity in this way, we obtained median lateral and axial fitting errors of 47.9 nm and 49.7 nm per frame. This frame rate regime favored diffusion up to ≈ $1\;\mu m^{2}\;s^{-1}$.

In contrast to sCMOS detectors, the EMCCDs in this work (Evolve 512 Delta, Teledyne) operated with an EM‐gain of 250 to maximize sensitivity and minimize read‐out noise. Like most EMCCDs, the shortest achievable exposure time with constant frame‐rate was 20 ms. While a shorter exposure time, or stroboscopic illumination, would ensure better sampling of fast molecular motion the absolute quantitation of molecular velocities is still limited by current detector speeds and sensitivities in a 3D context [38]. The design of brighter fluorescent probes would also facilitate faster temporal sampling. However, achieving higher temporal sampling requires greater illumination power density, which in turn increases the risk of phototoxic effects. Therefore, in this work, interpretations are drawn largely from relative features of protein motion, such as diffusive heterogeneity. These features provide metrics relevant to biological function that are unobtainable by alternative techniques, such as fluorescence recovery after photobleaching (FRAP) and fluorescence correlation spectroscopy (FCS), that also report on protein mobility. Further discussion of imaging quantities, temporal resolution and sensitivity can be found in Notes S3‐S5, and Refs [14, 15]. Importantly, without volumetric segmentation this observed heterogeneity in molecular motion is masked by the large population of calreticulin outside of ER inclusions.

## 3 Conclusion

In this work, we presented a simple optical method to enhance the sensitivity of 3D‐SMLM to molecular organization and diffusion measurements within subcellular structures. Our method involved utilizing FLFM in a widefield mode and a single‐molecule mode simultaneously, which enabled the segmentation of molecular localizations within organelles. We validated our methodology in living cellular systems through the detection of nuclear‐enrichment of soluble proteins, and by measuring heterogeneous molecular motion in the diseased ER. The accumulation of some false positive trajectories at the segmented boundary is a limitation of this approach. Future developments in camera technology and fluorescent proteins would improve the classification of false positives at organelle boundaries. Nonetheless, significant subcellular variation in diffusive motion was resolved, which demonstrates a pronounced sensitivity improvement in 3D‐SMT. Furthermore, while we presented our technique using two spectral channels to minimize fluorescence bleed‐through, in principle more channels could be accommodated through the optimization of emission filters to enable SMT across multiple sub‐cellular compartments.

We hope that spatially‐resolved SMLFM will provide access to intracellular biomolecular organization and dynamics in a manner akin to the impact TIRF microscopy has had on studies of the plasma membrane. Future developments to our optical approach could include (i) sub‐frame pulsed laser illumination to minimize phototoxic effects and improve temporal resolution [39], and (ii) the development of a spectral multiplexing pipeline to observe molecular diffusion within and between multiple subcellular volumes (e.g., viral entry into living cells) [40]. Future applications may span a wide range of biological disciplines, from tracking proteins at defined stages of the secretory pathway and diffusive measurements in transient subcellular environments, to probing organelle‐specific protein–protein interactions.

### 3.1 Statistics and Reproducibility

All statistical analyses were performed using a paired t‐test to assess significance between conditions. Data are presented as mean ± standard deviation unless otherwise stated. Significance thresholds of p* < 0.05, p** < 0.01, and p*** < 0.001 were used for t‐tests. Sample sizes (N) are indicated in the figure legends. Experiments were repeated independently at least three times to ensure reproducibility.

## Author Contributions

S.D., D.C.G., and S.F.L. conceived the project. D.C.G. and S.F.L. supervized the research. S.D. built the optical set‐up, performed all experiments and analyzed data. J.E.C. assisted in the design, implementation, and analysis of tracking experiments. B.F. wrote the deconvolution code base. C.J. assisted with the implementation of deconvolution code. J.D.M. assisted with early deconvolution methodologies. J.S.B. wrote and validated the pyDiffusion code base. S.J.M. acquired funding and provided critical feedback. S.D., D.C.G., and S.F.L. wrote the manuscript with input from all authors.

## Conflicts of Interest

The authors declare no conflicts of interest.

## Supporting information


**Supporting File 1**: smll71966‐sup‐0001‐SuppMat.pdf.

**Supporting File 2**: smll71966‐sup‐0002‐MovieS1.mp4.

## Data Availability

The data that support the findings of this study are openly available in a Zenodo repository at https://zenodo.org/records/15602782, reference number [15602782]
