# Supplementary material for: Volumetric Single‐Molecule Tracking Inside Subcellular Structures
Source: Small. 2026 Jan 15;22(9):e09162. doi: 10.1002/smll.202509162 (PMC12895227; doi:10.1002/smll.202509162)
Supplement: Supplementary file 1 — Supporting File 1: smll71966‐sup‐0001‐SuppMat.pdf. [file SMLL-22-e09162-s002.pdf]

Supplementary Information: Volumetric single-molecule tracking inside subcellular structures

Sam Daly[a,b], Joseph E. Chambers[b], Caroline Jones[a], Bin Fu[a], James D. Manton[c], Joseph S. Beckwith[a], Stefan J. Marciniak[b], David C. Gershlick[b,✉], and Steven F. Lee[a,✉]

[a]Yusuf Hamied Department of Chemistry, Lensfield Road, University of Cambridge, Cambridge, CB2 1EW, UK
[b]Cambridge Institute for Medical Research, University of Cambridge, Cambridge, CB2 0XY, UK
[c]MRC Laboratory of Molecular Biology, Francis Crick Avenue, Cambridge, CB2 0QH, UK

✉ e-mail: dg553@cam.ac.uk and sl591@cam.ac.uk

Contents

1 Materials and Methods **2**

2 Calreticulin diffusion is heterogeneous within ER inclusions **4**

3 Further validation—Single Molecule Tracking Channel **6**
3.1 Single molecule light field microscopy 6
3.2 Benchmarking diffusion analysis 6
3.3 Discussion of temporal resolution 6
3.4 Comparison with real-time volumetric SMT technologies 8

4 Further validation—Volumetric Organelle Channel **9**
4.1 Inclusion volume does not influence the diffusion coefficient 9
4.2 Inclusion brightness does not correlate with measured diffusion coefficient 9
4.3 ER inclusion dynamics were not affected by laser exposure 9
4.4 Comparison of volumetric reconstruction using diffusive and membrane-bound proteins 12

5 Notes on camera gain calibrations **13**

Supplementary Note 1: Materials and Methods

Plasmids

Two NLS sequences (PAAKRVKLD), incorporating a flexible linker (GGSGG) and EcoRV restriction site, were inserted into the vector backbone pEGFP-N1 (Clontech) and pHALO-N1 between the EGFP/HALO and MCS genes *via* a single-step KLD reaction. pHALO-C1 and pHALO-N1 were generated by replacing the eGFP in Clontech vectors with HALO using Gibson assembly (E2621L; New England Biolabs) as described previously [1]. Plasmids and primers used in this work are available upon reasonable request. All constructs were sequenced to verify their integrity.

Culture and preparation of HeLa cells

Hela cells were cultured at 37 °C and 5% CO_2, in Dulbecco's modified Eagle medium, DMEM (Gibco, 41966029), supplemented with 10% Fetal Bovine Serum (FBS; Sigma-Aldrich, F7424) and 0.2% MycoZapTM Plus-CL (Lonza, VZA–2012). Cells were passaged every three days and regularly tested for mycoplasma. For imaging, coverslips (631-0171, VWR) were cleaned under argon plasma (PDC-002, Harrick Plasma, Ithaca, NY) for 1 hour, transferred to 35 mm diameter 6-well culture dishes, and incubated with Matrigel for 1 hour (1.5 mL, 1:100 in cDMEM; Corning, 354277). Cells were seeded at a density of 0.1×10^3 cells cm^{-2} into fresh cDMEM and left overnight. For light-field and confocal microscopy of nuclei, cells were transfected with expression vectors encoding an NLS-EGFP fusion protein (2 µg DNA) at a 1:6 ratio of DNA to FuGene (µL). For single-molecule light-field microscopy, cells were transfected with expression vectors encoding an NLS-EGFP fusion protein (1.8 µg DNA) and a NLS-HaloTag fusion protein or HaloTag protein (0.2 µg DNA) at a 1:6 ratio of DNA to FuGene (µL, Promega). After 2 hours, the media was replaced with fresh cDMEM.

Culture and preparation of CHO cells

Chinese Hamster Ovary (CHO) cells (Clontech) were cultured in F12 Ham's nutrient mixture (Merck, Germany) supplemented with 10% FBS and 2 mM GlutaMAX (Thermo Fisher Scientific, USA) at 37 °C, 5% CO_2. For imaging, coverslips (631-0171, VWR) were cleaned under argon plasma for 1 hour and cells were seeded at a density of 1.04 $\times 10^3$ cells cm^{-2} in 35 mm diameter 6-well culture dishes. 6 hours after seeding, cells were transfected with expression vectors encoding an mEmerald-A1AT fusion protein (0.5 µg DNA) and a HaloTag-Calreticulin fusion protein (0.2 µg DNA), at a 1:4 ratio of DNA (µg) to lipofectamine LTX (µl), as per the manufacturer's instructions (Life Technology, UK). Expression vectors were reported previously in [2].

Fluorescence labeling

Immediately before imaging, cells expressing HaloTag fusion proteins were washed with phosphate buffered saline (PBS; 1 × 10 mL; 14040133, ThermoFisher), labeled with PA-JF_{646} HaloTag ligand (500 nM, Janelia Materials) in OptiMEM (10149832, Gibco) for 1 minute, then washed again with PBS (5 × 10 mL) and returned to culture medium (1 × 2 mL). When required for imaging, the coverslip was transferred into an AttoFluor cell chamber (A7816, ThermoFisher), the buffer replaced with FluoroBrite (1 mL w/ 25 mM HEPES, A1896701, ThermoFisher), and transferred to a temperature controlled microscope chamber (37 °C). Samples were imaged no longer than 2 hours after labeling.

Preparation of fluorescent bead coverslips

Coverslips (631-1570, VWR) were cleaned under argon plasma for 1 hour, incubated with poly-L-lysine (PLL; 50 µL; P4832, Sigma-Aldrich) for 10 minutes, and were washed with PBS (3 × 50 µL). Fluorescent beads (0.2 µm or 4 µm; F8807 and F8859, ThermoFisher) were diluted in PBS to a concentration of 3.4×10^7 particles mL^{-1}. The bead solution (50 µL) was then incubated on the PLL-coated coverslips for 3 minutes, which were then washed with PBS (3 × 50 µL) and transferred to the microscope for imaging.

Single-Molecule Light-Field Microscopy optical platform

The SMLFM platform described in this work was constructed using an epi-fluorescence microscope (Eclipse Ti–U, Nikon) housing a 1.27 NA water immersion objective lens (Plan Apo VC 60×, Nikon, Tokyo, Japan). Excitation was achieved using 640 nm (~1 kW cm^{-2} power density, iBeam Smart-S 640-S, Toptica, Munich, Germany), 488 nm (~40 W cm^{-2} power density, Cobolt), and 405 nm (~4 W cm^{-2} power density, iBeam Smart-S 405-S, Toptica, Munich, Germany) lasers, which were circularly polarized, collimated and focused on to the back focal plane (BFP) of the objective.

Unless stated otherwise, samples were excited with a highly inclined and laminated optical sheet (HILO) which was achieved by laterally displacing the excitation beam towards the edge of the BFP of the objective. The z-position of the objective was controlled with a scanning piezo (P–726 PIFOC, PI, Karlsruhe, Germany). Fluorescence was collected by the same objective and separated from the excitation beam using a quad-band dichroic mirror (Di01-R405/488/561/635-25×36, Semrock). The Fourier lens (f = 175 mm, ThorLabs) was placed in a 4f configuration with the tube lens (f = 200 mm, Nikon) to relay the conjugate BFP outside of the microscope body. The collimated emission beam path was then split chromatically at 90° using a long-pass dichroic (Di02–R635–25×36, Semrock). In the far-red emission path, a hexagonal microlens array (f = 175 mm, pitch = 2.39 mm) was placed in the BFP to relay the image plane onto an EMCCD (Evolve Delta 512, Photometrics, Tucson, AZ). Long-pass (BLP01-647R-25, Semrock) and band-pass (FF02-675/67-25, Semrock) emission filters were placed immediately before the detector to isolate fluorescence emission. In the short wavelength emission path, a hexagonal microlens array (f = 175 mm, pitch = 2.39 mm) was placed in the BFP within a rotation mount (RSP1/M, Thorlabs) to relay the image plane onto an EMCCD (Evolve Delta 512, Photometrics, Tucson, AZ) with radial alignment. Long-pass (BLP01-488R-25, Semrock) and band-pass (FF01-510/84-25, Semrock) emission filters were placed immediately before the detector to isolate fluorescence emission. The pixel size in image space was measured at 266 nm using a Ronchi ruling, which revealed no detectable spherical aberration.

Reconstruction of 3D-SMLM and SMT data

All experimental data were recorded as .tif stacks. 2D gaussian fitting of all emitter positions in all perspective views was carried out in Fiji using PeakFit (GDSC SMLM 2.0) to yield a set of 2D localizations for each raw frame. Given this initial set of 2D localizations, individual emitters were localized in 3D using custom MATLAB scripts available at [3] as outlined in [4]. For SMT analysis, a custom-written MATLAB code was used to temporally group localisations into single trajectories (x,y,z and t), as described previously [3]. Briefly, a minimum track length of 8 points was accepted using a search radius of 500 nm and allowing up to 1 consecutive dark frame. The subsequent datasets were analyzed using publicly available Python scripts at `https://github.com/TheLeeLab/pyDiffusion_LeeLab`. These were used to extract diffusion coefficients *via* mean square displacement analysis [5, 6] and 3D jump distances. Dark frames were accounted for through interpolation, which averaged the surrounding two tracking points to estimate the unknown position.

Axial calibration for SMLFM

Fluorescent beads (200 nm, Deep Red FluoSpheres, ThermoFisher) were immobilized on a glass slide and imaged to calibrate for deviations in experimental and calculated the disparity from the SMLFM optical model. Glass slides were cleaned under argon plasma (PDC-002, Harrick Plasma, Ithaca, NY) for 1 hour and incubated with poly-L-lysine (PLL, 50 µL, 0.1% w/v, Sigma-Aldrich, P820) for 10 minutes. Glass slides were washed with PBS (3 × 50 µL) and incubated with fluorescent beads (50 µL, *ca.* 3.6×10^8 particles mL^{-1}) for 3 minutes before washing further with PBS (3 × 50 µL). The piezo stage (P-726 PIFOC, PI, Karlsruhe, Germany) was used to scan the objective lens axially over 8 µm recording 10 frames at 30 ms exposure per 60 nm increment. The data was reconstructed in 3D and plotted against the known movement of the piezo stage. A linear fit was applied to the calibration curve, the gradient of which was a correction factor subsequently applied to all reconstructed data presented in this work.

Richardson-Lucy deconvolution

A custom MATLAB code was developed to perform RL deconvolution. The point spread function (PSF) was simulated over a depth range from z = –5 µm to z = 5 µm, covering the whole depth of field of the imaging system. The PSF model used in deconvolution adapted the approach described in [4]. To better account for spherical aberration caused by refractive index mismatch, the simulated PSF was combined with experimental data to generate a hybrid PSF [7]. Voxel dimensions were set to 266 nm isotropically, matching the experimental back-projected pixel size of imaging system, and ensuring consistent spatial sampling between the image and the reconstructed volume. All reconstructions were performed on a PC with an Intel i9-10900 processor and 64 GB of RAM.

Confocal microscopy

Volumes were acquired using a Zeiss LSM980 scanning confocal microscope with GaAsP detectors and a 63× 1.4 NA oil immersion objective. Samples were held at 37 °C in cDMEM (substituted with 25 mM HEPES) during the imaging process. Images were captured using bidirectional scanning over a 512 × 512 frame size with a 10 ms dwell time per pixel. The back-projected pixel size was 70 nm laterally and 240 nm axially in accordance with Nyquist sampling. The 488 nm laser power density was ~2 W cm^{-2} and an average of 43 images were collected per z-stack.

Supplementary Note 2: Calreticulin diffusion is heterogeneous within ER inclusions

Diffusion analysis data from Figure 4 has been reproduced here on a cell-to-cell basis. The same general trend to the repeat-to-repeat data is observed but greater variability in diffusion coefficients (Supplementary Figure 1a) and jump distances (Supplementary Figure 1b) reflects a disproportionate number of trajectories observed between cells and hence are likely sampling artifacts (Supplementary Figure 1c). Therefore, we chose to focus on replicate level variability.

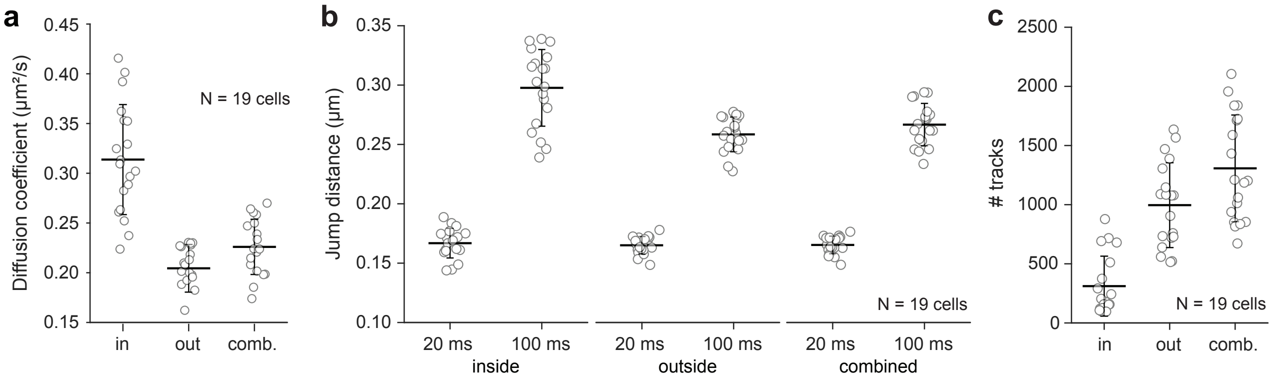

Supplementary Figure 1. Reproduction of Figure 4 on a cell-by-cell basis. a Average diffusion coefficient per cell inside and outside of measured ER inclusion volumes. Each data point represents the average value across the entire population of tracks within one of N = 19 cells. **b** Average jump distance (over 20 ms and 100 ms intervals) per cell both inside and outside of measured ER inclusion volumes. **c** Number of trajectories inside and outside of measured ER inclusions per cell.

Likewise, the distributions of calreticulin jump distances (JD) at 20 ms and 100 ms lag times are presented on a track-by-track basis as histograms in Supplementary Figure 2. At a lag time of $\Delta t = 20$ ms, the JD histograms for both populations are similar, suggesting comparable short-range diffusive behaviour (Supplementary Figure 2a & b). This observation is corroborated by the overlap in their cumulative distribution functions (CDFs). Although the JD_{in} population is smaller than JD_{out}, the spread of JDs is comparable, indicating that both populations of motion are adequately sampled.

At longer time lag, $\Delta t = 100$ms, the JDs of the two populations diverge markedly. A large immobile/highly confined fraction is observed for JD_{out}, with no clear change in the peak position alongside slight broadening of the distribution. These observations are consistent with the narrow, tubular geometry of the reticular ER, which restricts and adds directionality to displacement. In contrast, the distribution of JD_{in} values undergoes significant broadening, leading to a large tail and small confined population. The log-scale histograms and CDFs illustrate the differences well, with JD_{in} being right-shifted compared to JD_{out}, and a quantile difference at the 95th percentile of 0.19. These could suggest that calreticulin adopts a range of diffusive behaviours inside ER inclusions, reflecting a heterogeneous environment with variable macromolecular crowding and hence mobility. This is consistent with previous reports of molecular filtration in the ER after undergoing phase transition to a solid state [8]. Bulk fluorescence studies (*i.e.* FRAP) have been applied previously to study diffusion inside ER inclusions because single-molecule studies have been limited by the intrinsic 3D nature of these subcellular architectures that lie beyond the TIRF field.

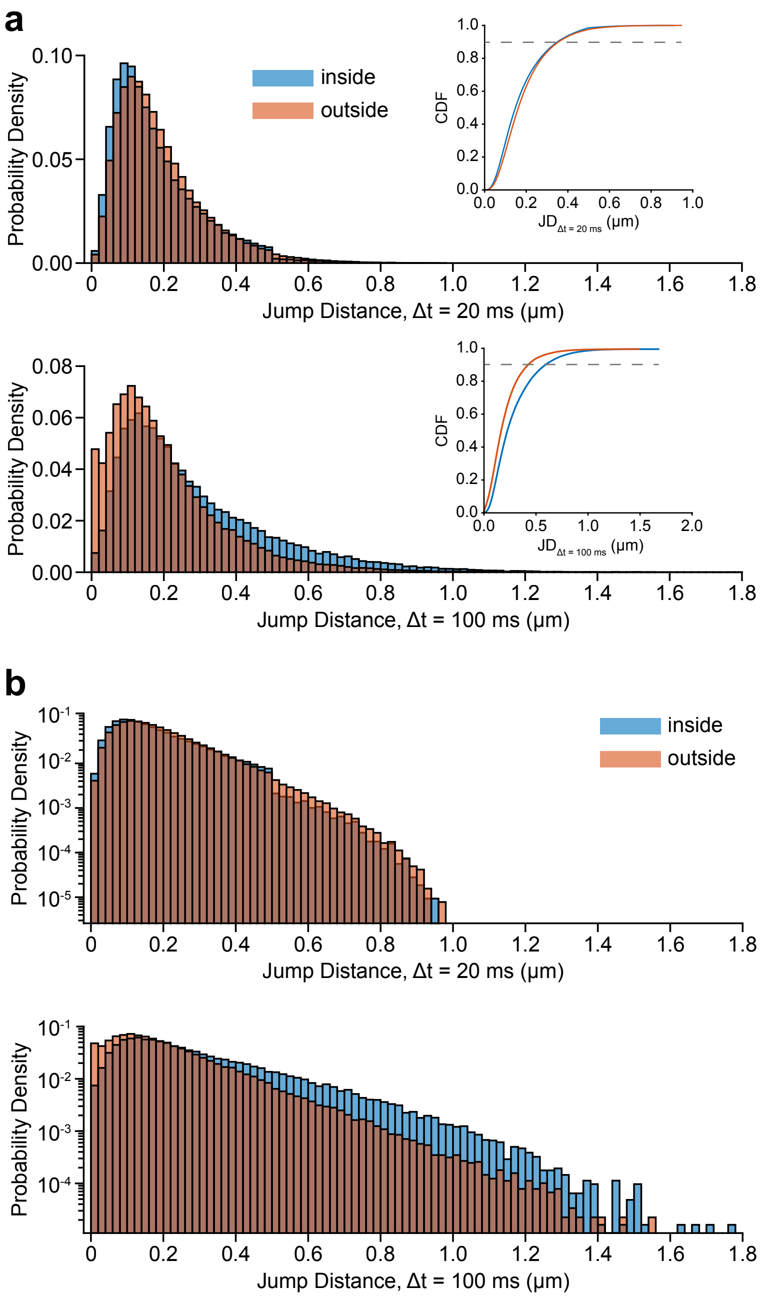

Supplementary Figure 2. Jump distance histograms for calreticulin. **a** Jump distance histogram (over a lag time of 20 ms or 100 ms) for calreticulin according to occurrence inside (blue) and outside (orange) of an ER inclusion. All tracks across N = 19 cells are shown. Insert shows the cumulative distribution function where the gray line indicates the 90th percentile. **b** Same as in **a** but on a log scale.

Supplementary Note 3: Further validation—Single Molecule Tracking Channel

3.1 Single molecule light field microscopy

Single molecule light field microscopy (SMLFM) was implemented to track the diffusive motion of the endoplasmic reticulum (ER) chaperone protein, calreticulin, alongside the instantaneous volumetric segmentation of tracks using Z-α_1-antitrypsin-mEmerald. A total of 19 regions of interest (ROIs) were acquired with an exposure time of 20 ms and comprised 20,000 frames, which is equivalent to a duration of ~7 minutes. A representative volumetric localization dataset comprising 80,000 localizations is presented in Supplementary Figure 3a. A representative median fitting error of 47.9 nm laterally and 49.7 nm axially was acquired, as shown in Supplementary Figures 3b & c. These correspond to a median of five perspective views used for fitting each 3D localization from raw 2D localization datasets for a median of 1147 detected photons per frame, see Supplementary Figure 3d & e. These values are consistent across all 19 ROIs and were acquired using the excitation and emission regime presented in Supplementary Figure 4 in a HILO illumination geometry.

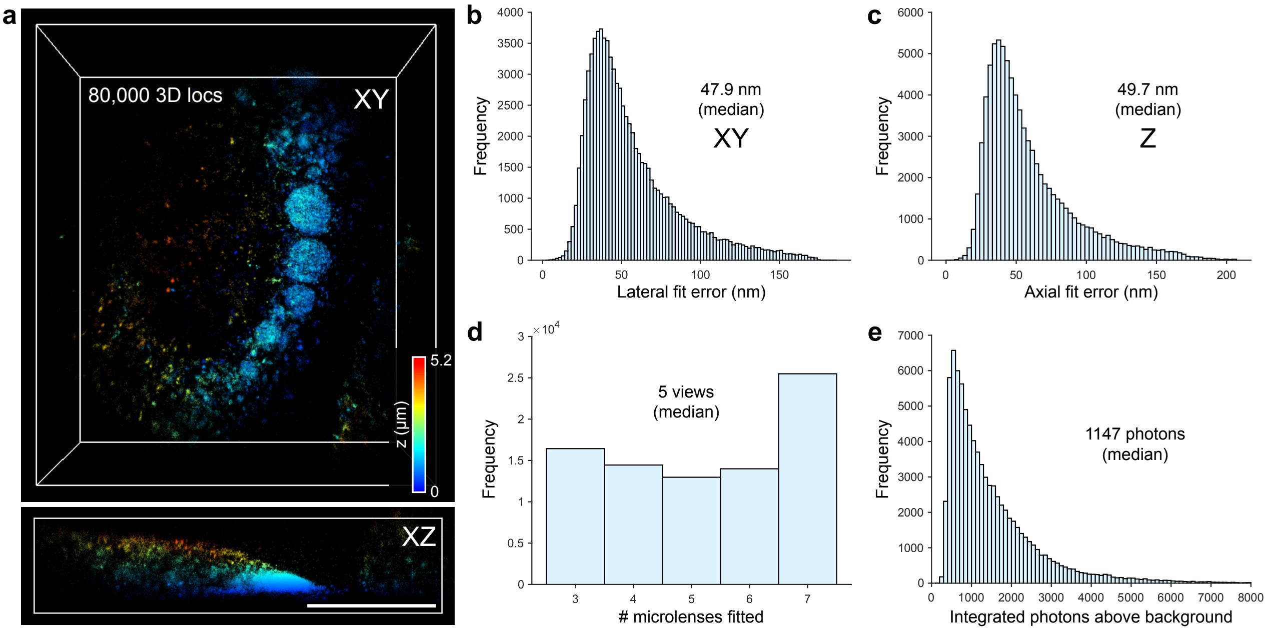

Supplementary Figure 3. Representative localization metrics. **a** A representative volumetric SMLM reconstruction from 20,000 frames comprising 80,000 points across a 5.2 µm depth-of-field. Scale bar is 10 µm. **b** Corresponding lateral (XY) and **c** axial (Z) fitting error per frame evaluated from 3D reconstruction. **d** Proportion of 3D localizations reconstructed from 3–7 microlenses. **e** Fluorescent photons detected above background per 3D localization per frame.

3.2 Benchmarking diffusion analysis

Mean square displacement (MSD) analysis was conducted on realistic diffusion simulations, see Supplementary Figure 5, and retrieved the expected diffusion coefficient values with assumed motion blur.

3.3 Discussion of temporal resolution

In order to accurately quantify absolute diffusion, SMT experiments require sufficient temporal resolution to sample fast and slow trajectories. Much work has been done to overcome the need for fast camera acquisition [9–11]. Generally, 2D-SMT experiments can afford faster acquisition rates compared to 3D-SMT, for which higher sensitivity detection is necessary due to the incorporation of complex optical components that reduce photon throughput. Nonetheless, even for a comparatively short 2D exposure time of 5 ms [12] diffusive motion above 1 $\mu m^2 s^{-1}$ is still undersampled, see Supplementary Table 1.

In this work we report relative diffusion metrics rather than absolute values to account for the undersampling of high speed motion. In SMLFM, a minimum of ~400 photons was required for 3D detection. Using sensitive EMCCDs with a minimum exposure time of 20 ms enabled the detection of 1000s of trajectories per cell with volumetric spatial precision below 50 nm across 19 cells and 24,836 tracks.

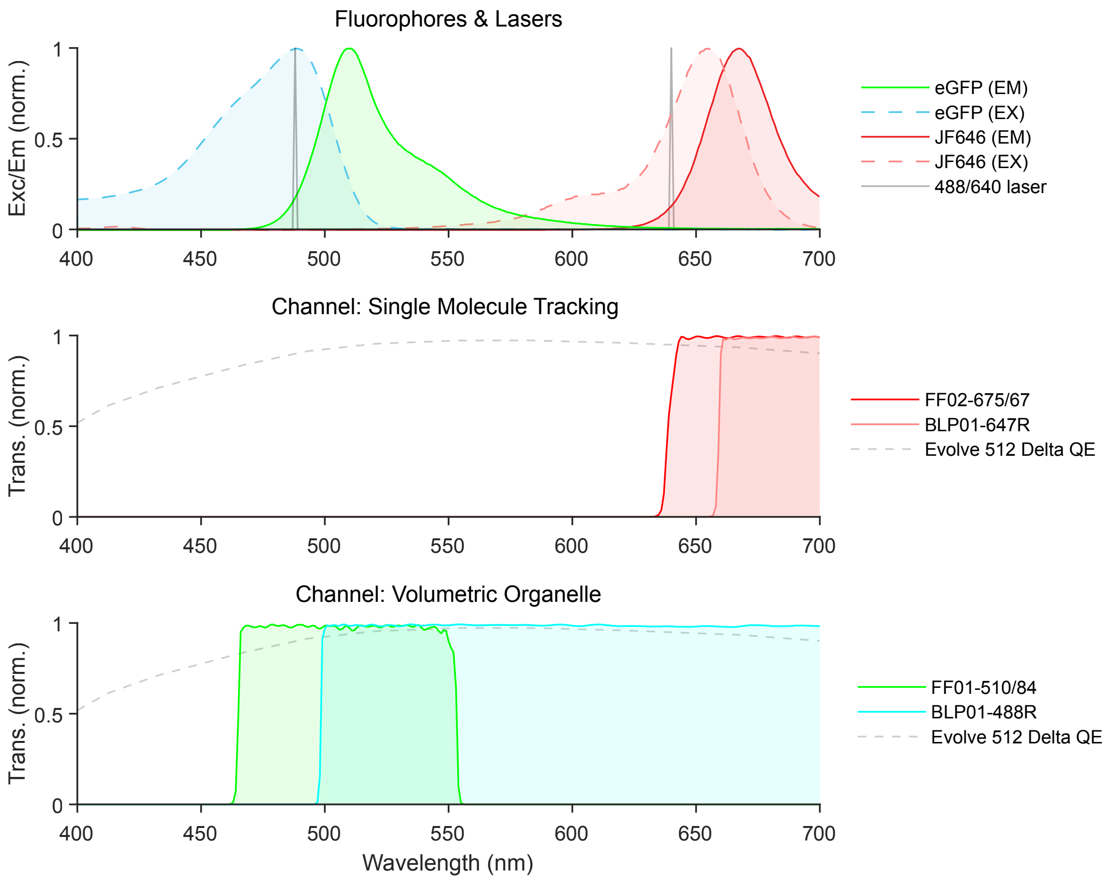

Supplementary Figure 4. Optical properties of fluorophores and filters. Normalised absorbance (dashed line) and emission (solid line) spectra for eGFP and JF646 used in this work. Excitation and emission filters are presented as a function of normalised transmission.

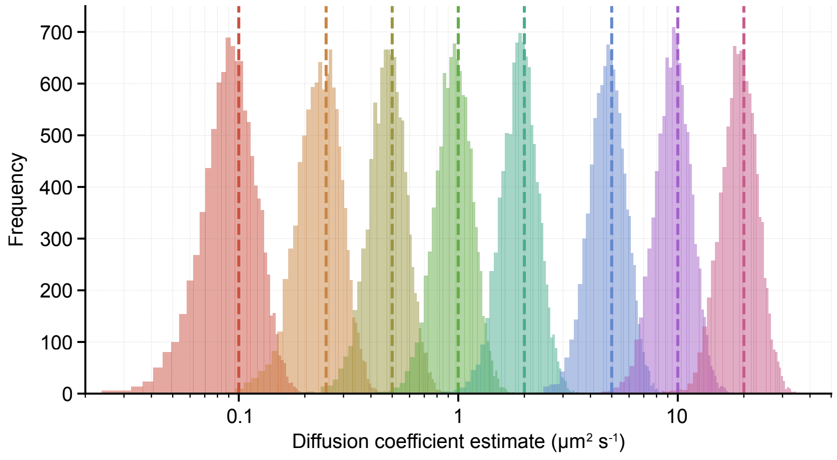

Supplementary Figure 5. Testing of MSD analysis code. Each color of histogram represents the estimation of a diffusion coefficient, D, from 10,000 individual trajectories (each containing 50 timesteps, displacements occurring in three dimensions) of a specified input diffusion coefficient (dotted lines). The code recapitulates the expected diffusion coefficient values. The displacements are simulated in accordance with Ref [6] assuming realistic motion blur (R=1/6, see Equation 5 of reference), a PSF with a 250 nm standard deviation and a localization precision per point of 50 nm. The timesteps of the displacements are assumed to be 1/10th of the diffusion coefficient (times micron squared) or 20 ms, whichever value was larger.

Supplementary Table 1. Maximum frame interval Δt (ms) for different 3D diffusion coefficients D and localisation precisions, using $\Delta t = \sigma^2/6D$.

D (μm^2/s)	Localisation Precision (nm)						
	10	20	30	40	50	75	100
0.001	17	67	150	267	417	938	1667
0.01	1.7	6.7	15	27	42	94	167
0.1	0.17	0.67	1.5	2.7	4.2	9.4	17
1	0.017	0.067	0.15	0.27	0.42	0.94	1.7
10	0.0017	0.0067	0.015	0.027	0.042	0.094	0.17
100	0.00017	0.00067	0.0015	0.0027	0.0042	0.0094	0.017

3.4 Comparison with real-time volumetric SMT technologies

Laser scanning confocal microscopy combined with a SPAD array and cylindrical lens can enable volumetric SMT alongside orthogonal measurements, such as lifetime [13]. A real-time feedback loop keeps the particle centred while its motion is tracked until it either escapes the volume or becomes photobleached. A typical spatial precision of 40 nm laterally and 60 nm axially was obtained for 100 photons. Through more complex beam shaping, MINFLUX tracking improves precision to ~3 nm in 3D [14, 15]. We have previously demonstrated <28 nm spatial precision in 3D for 2,500 photons using SMLFM. We now show that the instantaneous capture of subcellular volumes alongside high-density tracking allows for spatially selective tracking within subcellular structures.

Two clear advantages of SMLFM are its throughput and ease of implementation. Confocal SMT requires hardware for high-speed mechanical timing/detectors and MINFLUX also requires additional complex beamshaping optics. Both MINFLUX and confocal SMT possess a very low comparative throughput of one particle at a time, while SMLFM affords an optical redundancy through repeated measurement of single molecule fluorescence enabling localization densities of 0.3 μm^{-3} or above per frame, which can result in 1000s of trajectories in minutes. Therefore, confocal SMT and MINFLUX demand extremely sparse localization datasets to operate necessitating non-physiological labelling strategies. Confocal and MINFLUX typically integrate lower background fluorescence (a few photons) compared to our approach which integrates background across the whole excitation volume. In practice inclined illumination can reduce this to ~10 photons per pixel. However, MINFLUX is also more sensitive to background fluorescence and residual intensity within the excitation doughnut.

In addition to high-throughput tracking and facile implementation into existing widefield microscopes, the simultaneous volumetric segmentation of subcellular organelles is possible with SMLFM. The bulk segmentation of subcellular features is restricted to 2D for confocal and MINFLUX and hence these techniques neglect precise axial segmentation. The strength of confocal and MINFLUX stems from high temporal resolution (<1 ms) and trajectory duration (0.1–2 s) for the capture of fast diffusive motion. In this work we demonstrate our optical approach using EMCCDs with a minimum exposure time of 20 ms. However, as the read noise of sCMOS cameras improves to below 1 electron, the possibility of much faster acquisitions will become possible. Ultimately, optimal sensitivity is obtained with single element detectors (*i.e.* SPAD/PMT), but this comes at the cost of lower throughput and hence loss of sensitivity to rare events.

Supplementary Note 4: Further validation—Volumetric Organelle Channel

4.1 Inclusion volume does not influence the diffusion coefficient

To confirm that segmentation does not influence the diffusion coefficient measurements in Figure 4, the inclusion volume and the diffusion coefficient of the tracks it contains should not be correlated. This relationship was evaluated and is presented in Supplementary Figure 6. Here, the diffusion coefficient was evaluated as a function of the surrounding inclusion volume, which revealed no trend. This is confirmed by a Pearson correlation coefficient of effectively zero and p-value > 0.05, see Supplementary Table 2.

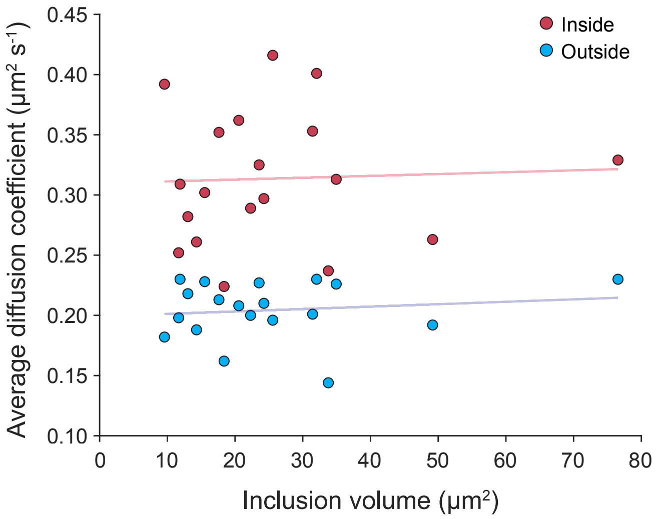

Supplementary Figure 6. Diffusion coefficient *vs.* inclusion volume. Inclusion volume was evaluated per field-of-view and is plotted here against the average diffusion coefficient of tracks. No trend is observed, which suggests that segmentation does not influence the diffusion coefficient.

Supplementary Table 2. Pearson correlation coefficient analysis for Supplementary Figure 6.

	**PCC**	**p-value**
Outside	0.13	0.59
Inside	0.04	0.86

4.2 Inclusion brightness does not correlate with measured diffusion coefficient

We evaluated the relationship between the brightness of Z-α_1-antitrypsin-mEmerald and average diffusion coefficient of the trajectories inside the ER inclusion to investigate whether polymer density likely influenced diffusion dynamics. No correlation was observed, see Supplementary Figure 7, which suggests that either there is likely no measurable relationship between polymer density and diffusion dynamics, or that the assumption that brightness corresponds to polymer density is not suitable.

4.3 ER inclusion dynamics were not affected by laser exposure

Volumetric snapshots across the imaging duration revealed that ER inclusions range in number between cells and are morphologically dynamic. Inclusions were observed to be slowly mobile with displacements along the order of ~1 μm across the 20,000 frames. In some cases smaller inclusions were also observed to bud-off from, or fuse with, larger inclusions as shown in Supplementary Figure 8.

Laser illumination did not induce changes in ER inclusion dynamics. To evaluate this, the difference in the average diffusion coefficient across the first and last 2000 frames was evaluated. A value of 0.000680 $\pm$ 0.036 $\mu m^2\,s^{-1}$ was determined, which confirmed that the average observed diffusion was unaffected by laser exposure. Next, the total volume of each ER inclusion was analysed as a function of laser irradiation duration (whilst also accounting for their

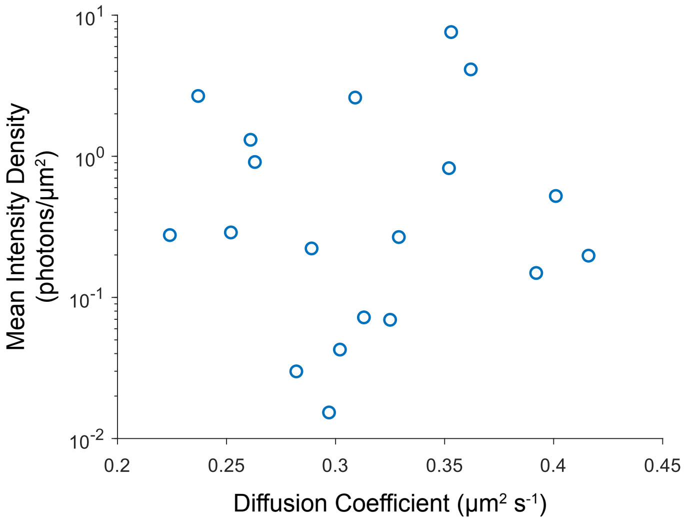

Supplementary Figure 7. Diffusion coefficient *vs.* inclusion brightness. No correlation was observed between the brightness of an ER inclusion and the average diffusion coefficient within. Each point represents an individual cellular repeat.

morphological changes over time). The results of this are shown in Supplementary Figure 9, which shows no change in the total volume of ER inclusions across the course of imaging. Slight variations in the measured volume across a given time trace can be explained by size and intensity filtration when classifying ER inclusions. For example, if a small inclusion were to bud-off from a larger inclusion, its brightness might fall below the threshold criteria for 3D reconstruction. These experimental conditions and results are consistent with previous studies, which also supports our claim that the differences in molecular motion inside and outside of inclusions are not induced by the experimental conditions.

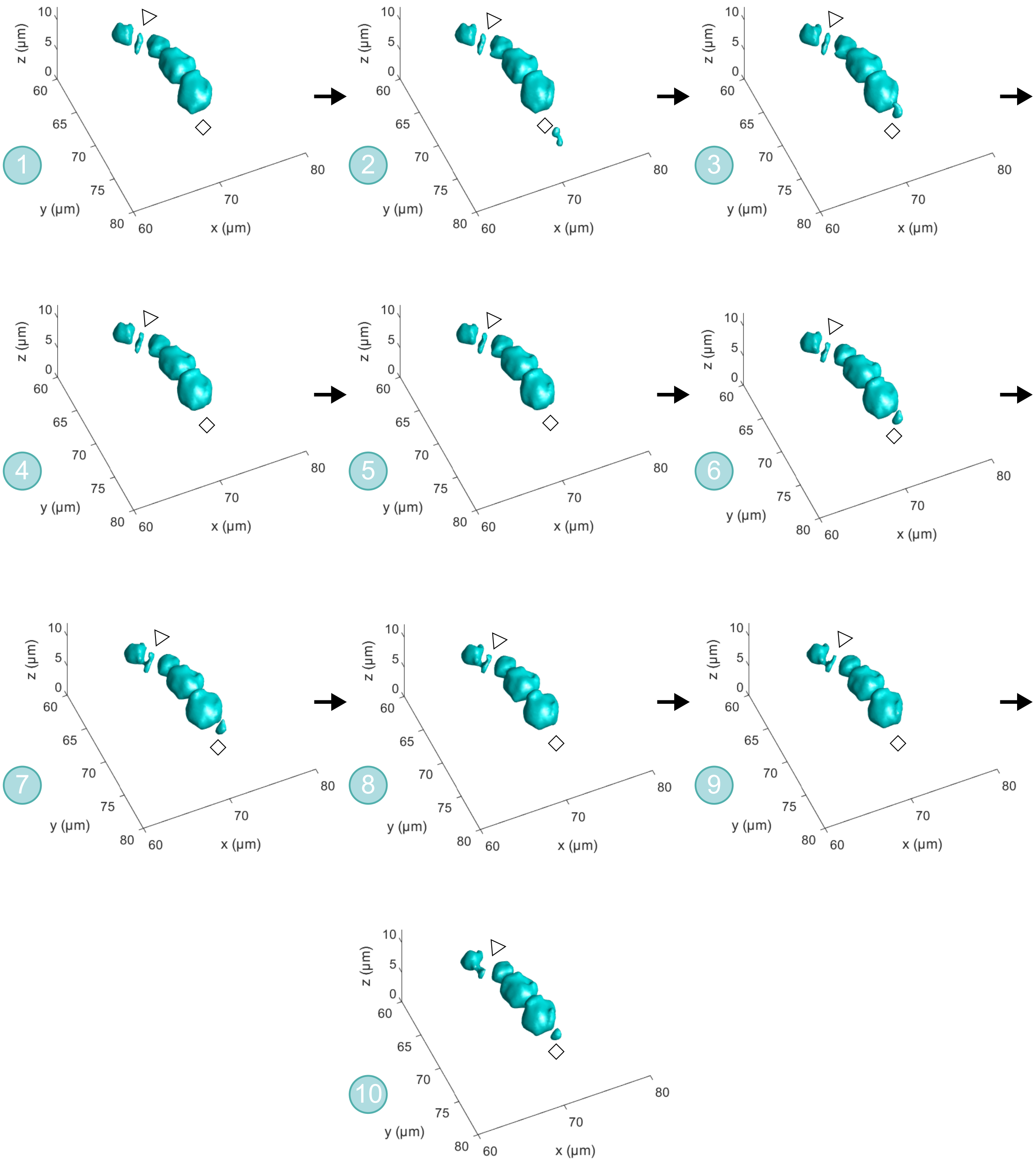

Supplementary Figure 8. Morphological evolution of ER inclusions over time. Volumetric reconstruction of ER inclusions from a representative ROI across the whole acquisition (20,000 frames). To improve SNR for each volumetric reconstruction, raw fluorescence images were temporally averaged across 2000 frames prior to Richardson-Lucy deconvolution. The triangle indicates fusion between two inclusions of different size. The rhombus indicates a representative small, dim inclusion that is at the threshold boundary for volumetric reconstruction.

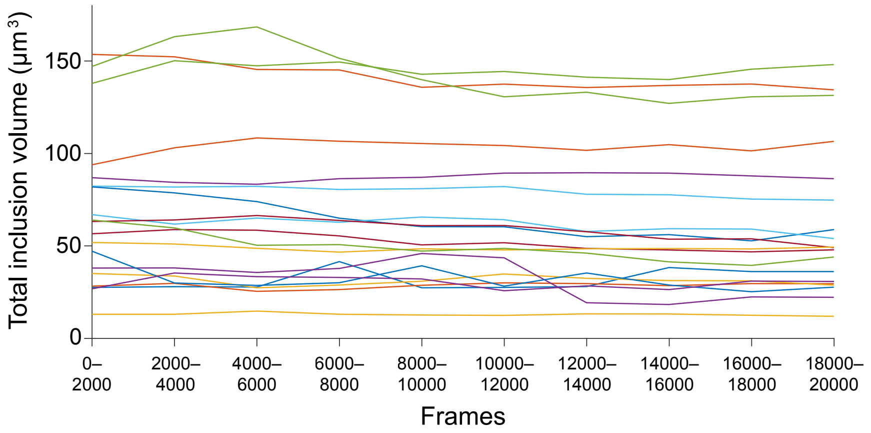

Supplementary Figure 9. Total inclusion volume *vs.* time. Cumulative volume of all inclusions per field-of-view averaged over 2000 frame bins (N = 19 cells) to improve SNR.

4.4 Comparison of volumetric reconstruction using diffusive and membrane-bound proteins

In this work, volumetric segmentation was validated in a biological context through the SMT of the diffusive protein, calreticulin. Volumetric segmentation was achieved alongside tracking using Z-α_1-antitrypsin fused to mEmerald, another diffusive protein marking ER inclusions. Unlike diffusive proteins, VO imaging using membrane-localized proteins can be more sensitive to photobleaching due to a smaller protein pool.

Here, we demonstrate VO imaging using Golgi-localized membrane protein, GalT fused to GFP. Cells were imaged for 30000 frames (approx. 10 minutes) under an identical excitation regime as that of Z-α_1-antitrypsin, see Supplementary Figure 10a & b. The fluorescence intensity of GalT-GFP was observed to have decreased by 51% after 30000 frames relative to the start of imaging, see Supplementary Figure 10c. After 20000 frames—the duration over which Z-α_1-antitrypsin was imaged in this work—fluorescence intensity had decreased by 33% from photobleaching while no significant change was observed for Z-α_1-antitrypsin-mEmerald, see Supplementary Figure 10d. Despite a 33% loss of fluorescence signal across 20000 frames, volumetric reconstructions of GalT-GFP appeared to remain largely unaffected by the reduced SNR, see Supplementary Figure 10e. Since the prolonged exposure of cells is unfavourable due to the risk of phototoxicity, this demonstration confirms that volumetric segmentation with FLFM is amenable to both diffusive and membrane-localized proteins.

In principle, the temporal averaging of fluorescent signal in the VO channel prior to volumetric reconstruction could significantly improve the resulting volume if cellular motion is comparatively slow compared to diffusion dynamics.

Supplementary Note 5: Notes on camera gain calibrations

Conversion gain describes the relationship between counts observed in raw imaging data and the number of photons incident on the detector. The conversion gain for the EMCCDs used in this work was quantified with publicly available code (`https://github.com/TheLeeLab/cameraCalibrationCMOS`) using the method described in [16].

Full sensor images (512×512 pixels) were recorded for 2000 frames at six exposure times (10, 20, 40, 80, 160, 320 ms), including dark frames, with electron-multiplying (EM) gain set to a value of 1. The detector was the re-calibrated with an EM gain of 250, which is the value utilized in all imaging experiments described in this work. The quantum efficiency of 0.95 at 680 nm was taken into account. Conversion gain was calculated *via* the following equation:

$$\text{conversion gain} = \text{gain (with EM gain set to 1)} \times \text{EM gain} \tag{1}$$

which gave values of 42 (VO channel) and 40 (SM channel) counts/photoelectron. At an EM gain of 250, average read noise was observed to be ~1 electron. The in-built 'Rapid-Cal' feature of the Evolve 512 Delta ensured that the total 'conversion' gain for all experiments was consistent despite aging of the EMCCD register.

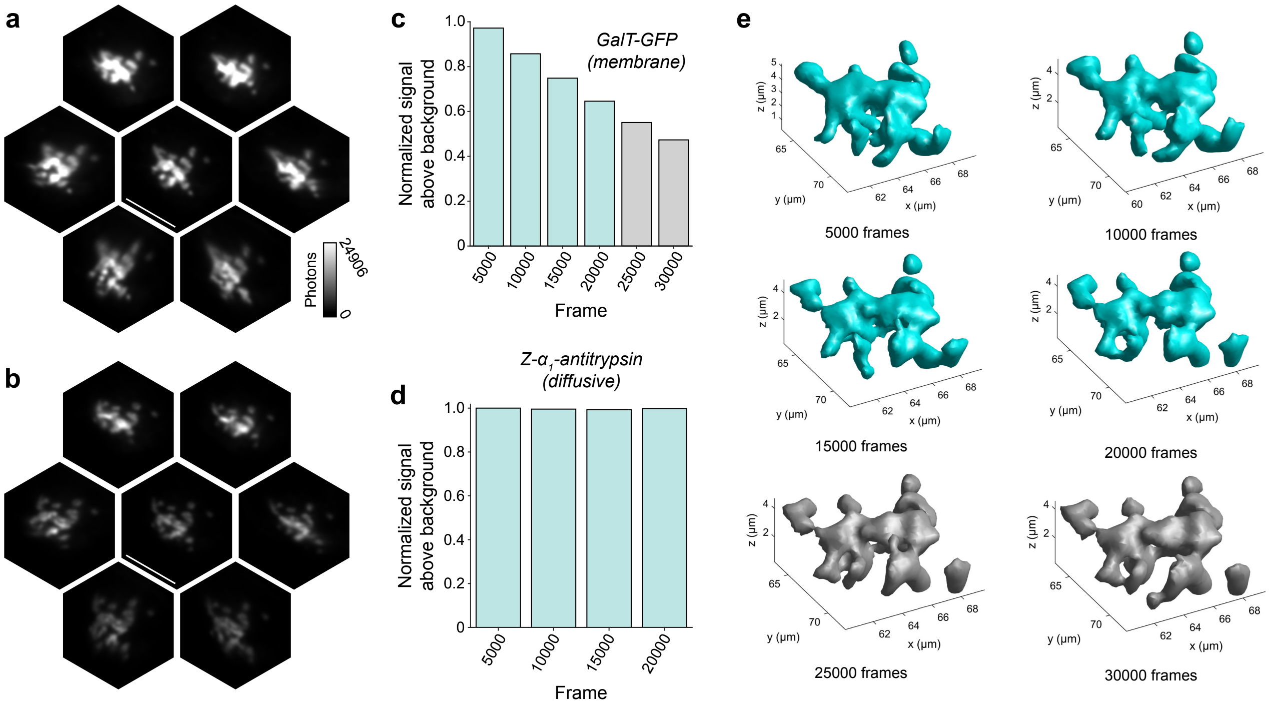

Supplementary Figure 10. Effect of photobleaching on volumetric organelle imaging. **a** Background-subtracted FLFM image of GalT-GFP averaged across frames 1–5000 and **b** frames 25000–30000. Scale bar is 10 µm. **c** Volumetric reconstruction of GalT-GFP signal at 5000 frame intervals *via* Richardson-Lucy deconvolution. **d** Normalized mean fluorescence signal of GalT-GFP above background across the course of 30000 frames. A 33% reduction in fluorescence intensity was observed between 1 and 20000 frames, which is the duration of laser exposure used in this work. **e** Normalized mean fluorescence signal of diffusive protein, Z-α_1-antitrypsin-mEmerald above background across the course of 20000 frames.

Supplementary References

1. Conceição Pereira, Danièle Stalder, Georgina S. F. Anderson, Amber S. Shun-Shion, Jack Houghton, Robin Antrobus, Michael A. Chapman, Daniel J. Fazakerley, and David C. Gershlick. The exocyst complex is an essential component of the mammalian constitutive secretory pathway. *The Journal of Cell Biology*, 222(5):e202205137, May 2023.
2. Alexander Y. Maslov, Sergey Makhortov, Shixiang Sun, Johanna Heid, Xiao Dong, Moonsook Lee, and Jan Vijg. Single-molecule, quantitative detection of low-abundance somatic mutations by high-throughput sequencing. *Science Advances*, 8(14):eabm3259, April 2022.
3. Sam Daly, João Ferreira Fernandes, Ezra Bruggeman, Anoushka Handa, Ruby Peters, Sarah Benaissa, Boya Zhang, Joseph S. Beckwith, Edward W. Sanders, Ruth R. Sims, David Klenerman, Simon J. Davis, Kevin O'Holleran, and Steven F. Lee. High-density volumetric super-resolution microscopy. *Nature Communications*, 15(1):1940, March 2024.
4. Ruth R. Sims, Sohaib Abdul Rehman, Martin O. Lenz, Sarah I. Benaissa, Ezra Bruggeman, Adam Clark, Edward W. Sanders, Aleks Ponjavic, Leila Muresan, Steven F. Lee, and Kevin O'Holleran. Single molecule light field microscopy. *Optica*, 7(9):1065–1072, September 2020.
5. Xavier Michalet. Mean square displacement analysis of single-particle trajectories with localization error: Brownian motion in an isotropic medium. *Physical Review E*, 82(4):041914, October 2010. Publisher: American Physical Society.
6. Xavier Michalet and Andrew J. Berglund. Optimal diffusion coefficient estimation in single-particle tracking. *Physical Review E*, 85(6):061916, June 2012. Publisher: American Physical Society.
7. Xuanwen Hua, Wenhao Liu, and Shu Jia. High-resolution Fourier light-field microscopy for volumetric multi-color live-cell imaging. *Optica*, 8(5):614–620, May 2021. Publisher: Optica Publishing Group.
8. Joseph E. Chambers, Nikita Zubkov, Markéta Kubánková, Jonathon Nixon-Abell, Ioanna Mela, Susana Abreu, Max Schwiening, Giulia Lavarda, Ismael López-Duarte, Jennifer A. Dickens, Tomás Torres, Clemens F. Kaminski, Liam J. Holt, Edward Avezov, James A. Huntington, Peter St George-Hyslop, Marina K. Kuimova, and Stefan J. Marciniak. Z-α_1 -antitrypsin polymers impose molecular filtration in the endoplasmic reticulum after undergoing phase transition to a solid state. *Science Advances*, 8(14):eabm2094, April 2022.
9. Limin Xiang, Kun Chen, Rui Yan, Wan Li, and Ke Xu. Single-molecule displacement mapping unveils nanoscale heterogeneities in intracellular diffusivity. *Nature Methods*, 17(5):524–530, May 2020. Publisher: Nature Publishing Group.
10. Koen J. A. Martens, Bartosz Turkowyd, Johannes Hohlbein, and Ulrike Endesfelder. Temporal analysis of relative distances (TARDIS) is a robust, parameter-free alternative to single-particle tracking. *Nature Methods*, pages 1–8, January 2024. Publisher: Nature Publishing Group.
11. Megan A. Steves and Ke Xu. SpeedyTrack: Direct microsecond wide-field single-molecule tracking and super-resolution mapping via CCD vertical shift, April 2025.
12. Christopher J. Obara, Jonathon Nixon-Abell, Andrew S. Moore, Federica Riccio, David P. Hoffman, Gleb Shtengel, C. Shan Xu, Kathy Schaefer, H. Amalia Pasolli, Jean-Baptiste Masson, Harald F. Hess, Christopher P. Calderon, Craig Blackstone, and Jennifer Lippincott-Schwartz. Motion of VAPB molecules reveals ER–mitochondria contact site subdomains. *Nature*, 626(7997):169–176, February 2024.
13. Andrea Bucci, Giorgio Tortarolo, Marcus Oliver Held, Luca Bega, Eleonora Perego, Francesco Castagnetti, Irene Bozzoni, Eli Slenders, and Giuseppe Vicidomini. 4D Single-particle tracking with asynchronous read-out single-photon avalanche diode array detector. *Nature Communications*, 15(1):6188, July 2024.
14. Francisco Balzarotti, Yvan Eilers, Klaus C. Gwosch, Arvid H. Gynnå, Volker Westphal, Fernando D. Stefani, Johan Elf, and Stefan W. Hell. Nanometer resolution imaging and tracking of fluorescent molecules with minimal photon fluxes. *Science*, 355(6325):606–612, February 2017.
15. Takahiro Deguchi, Malina K. Iwanski, Eva-Maria Schentarra, Christopher Heidebrecht, Lisa Schmidt, Jennifer Heck, Tobias Weihs, Sebastian Schnorrenberg, Philipp Hoess, Sheng Liu, Veronika Chevyreva, Kyung-Min Noh, Lukas C. Kapitein, and Jonas Ries. Direct observation of motor protein stepping in living cells using MINFLUX. *Science*, 379(6636):1010–1015, March 2023.
16. Fang Huang, Tobias M. P. Hartwich, Felix E. Rivera-Molina, Yu Lin, Whitney C. Duim, Jane J. Long, Pradeep D. Uchil, Jordan R. Myers, Michelle A. Baird, Walther Mothes, Michael W. Davidson, Derek Toomre, and Joerg Bewersdorf. Video-rate nanoscopy using sCMOS camera–specific single-molecule localization algorithms. *Nature Methods*, 10(7):653–658, July 2013. Number: 7 Publisher: Nature Publishing Group.
